# Gingival Augmentation Using Injectable Platelet-Rich Fibrin (i-PRF)—A Systematic Review of Randomized Controlled Trials

**DOI:** 10.3390/jcm13185591

**Published:** 2024-09-20

**Authors:** Jacek Żurek, Wojciech Niemczyk, Marzena Dominiak, Stanisław Niemczyk, Rafał Wiench, Dariusz Skaba

**Affiliations:** 1 Specialist Medical Practice, Polne Wzgórze 11 Street, 32-300 Olkusz, Poland; kontakt@jacekzurek.pl; 2 Department of Periodontal Diseases and Oral Mucosa Diseases, Faculty of Medical Sciences in Zabrze, Medical University of Silesia, Pl. Traugutta 2, 41-800 Zabrze, Poland; dskaba@sum.edu.pl; 3 Department of Dental Surgery Medical, University of Wroclaw, 50-425 Wroclaw, Poland; marzena.dominiak@umw.edu.pl; 4 Municipal Hospital No. 4 in Gliwice, Zygmunta Starego 20, 44-100 Gliwice, Poland

**Keywords:** platelet-rich plasma, platelet-rich fibrin, periodontics, blood platelets, percutaneous collagen induction, phenotype, gingiva, wound healing

## Abstract

**Background:** In recent years, the utilization of blood concentrates in dentistry has become increasingly prevalent. In 2014, the development of injectable platelet-rich fibrin (i-PRF) was achieved. One of the key benefits of i-PRF is its ability to consistently release a range of growth factors. This review aimed to determine whether i-PRF could be used for gingival augmentation. **Methods:** The research involved a search of the PubMed, Embase, Scopus, and Google Scholar databases using the following search terms: (“microneedling” or “micro needling” or “injectable platelet-rich fibrin” or “i-PRF”) and (“gingival augmentation” or “augmentation” or “attached gingiva” or “attached mucosa” or “soft tissue augmentation” or “KM” or “keratinized mucosa”). **Results:** Of the 668 results, 8 articles meeting the inclusion criteria were included in the article. The results of the studies analyzed indicated a significant increase in gingival thickness. Furthermore, some articles demonstrated an increase in keratinized tissue width. The augmentation of the gingival thickness with i-PRF yielded no inferior results in comparison to the free gingival graft, which is the current gold standard, resulting in a superior aesthetic outcome and a reduction in postoperative discomfort. **Conclusions:** This systematic review allowed the authors to conclude that the use of i-PRF or hyaluronic acid may be the first step towards developing a non-surgical method of gingival augmentation.

## 1. Introduction

### 1.1. Rationale—Biotype

In 1969, Ochsenbein and Ross initially proposed that there were two principal categories of gingival morphology: scalloped and thin or flat and thick gingiva [1]. The term ‘periodontal biotype’ was subsequently employed by Seibert and Lindhe, who distinguished between gingival tissue types as either thin–scalloped or thick–flat [2]. In contrast to the thick gingival tissues, which are relatively dense in appearance and have a rather wide zone of keratinized gingiva, the thin biotype is delicate and translucent, friable, and has a minimum zone of attached gingiva [3]. The response of gingival biotypes to inflammation, restorations, trauma, and parafunctional habits is more pronounced in the thin biotype [3,4]. The gingival thickness affects the treatment outcome, possibly because of the difference in the amount of blood supply to the underlying bone and susceptibility to resorption [4]. Furthermore, the thin biotype is also a significant risk factor for gingival recessions [5,6,7,8]. A systematic review by Zweers et al. revealed that the thin biotype is present in 42.3% of the population [9]. Furthermore, the gingival thickness diminished considerably with age in both arches [10]. The periodontal phenotype is defined as the combination of the gingival biotype (GB) and the thickness of buccal bone (bone morphotype) [11]. The gingival phenotype comprises two components: gingival thickness (GT) and keratinized tissue width (KTW). The classification of thick and GB was based on the measurement of GT, with a GT of 2 mm defining the thick GB and a GT of 1.5 mm or less defining the thin GB [12,13]. Nevertheless, there are many classifications of gingival thickness, and some define a thin phenotype only below a thickness of 1mm [14]. Some different approaches have been proposed for increasing the gingival thickness, including root coverage and non-root coverage techniques [15]. Among the root coverage procedures, we can distinguish between acellular dermal matrix (ADM), collagen matrix (CM), flap, and connective tissue graft (CTG), while non-root coverage procedures include ADM, apically positioned flap (APF), living cellular construct (LCC), CM, and free gingival graft (FGG). The current gold standard for increasing the amount of keratinized tissue is FGG. It provides stability over time, stops the progression of recession formation and helps patients maintain good hygiene. Its biggest drawbacks, on the other hand, are the considerable discomfort for the patient due to the additional interference in the donor site, and the poor aesthetic results [15,16,17].

### 1.2. Rationale—Molecular Factors

In recent years, the utilization of blood concentrates in dentistry has become increasingly prevalent. These autologous treatments have been demonstrated to facilitate natural healing, accelerate tissue regeneration, and provide patients with a more comfortable postoperative outcome [18,19,20,21,22,23]. A number of platelet-rich concentrates, including platelet-rich plasma (PRP) and platelet-rich fibrin (PRF), have been proposed and utilized for tissue regeneration in a variety of in vitro and in vivo studies [24,25,26]. Platelets play a pivotal role in the process of wound healing. Once activated, they secrete a range of factors that stimulate cell proliferation, including platelet-derived growth factor (PDGF), transforming growth factor (TGF-β), and insulin-like growth factor I (IGF-I). Furthermore, they secrete fibrin, fibronectin, and vitronectin, which form a matrix for connective tissue and facilitate the migration of cells by acting as adhesion molecules. Consequently, they play a pivotal role, in influencing processes such as cell proliferation, collagen synthesis, and osteoid formation [27]. The formation of new fibers results in the thickening of the tissue, a process known as neocollagenesis. Additionally, fibroblasts stimulate neoangiogenesis by promoting the proliferation of endothelial cells in the vessels. The remodeling of tissue persists from approximately eight weeks up to one year [28]. In 2014, the development of injectable platelet-rich fibrin (i-PRF) was achieved through the adjustment of spin centrifugation forces. The centrifugation of blood in non-glass centrifugation tubes at lower centrifugation speeds resulted in the formation of a flowable PRF product, designated i-PRF [29]. One of the key benefits of i-PRF is its ability to consistently release a range of growth factors, including PDGF, TGF-β, and IGF-I [30,31,32]. These growth factors, when released in a controlled and consistent manner, have the ability to promote cell migration by inducing the expression of essential proteins, such as type I collagen and transforming growth factors [33,34].

### 1.3. Objectives

The authors of this article observed a dearth of reviews on this topic, which motivated them to conduct a comprehensive review to ascertain the most reliable findings. This article aimed to assess the potential of i-PRF as a preliminary approach to non-surgical gingival tissue augmentation.

## 2. Material and Methods

### 2.1. Focused Question

A systematic review was conducted in accordance with the PICO framework [35], as follows: In patients with the thin gingival biotype (Population), can platelet-rich fibrin with microneedling (Intervention) serve as an alternative method (Comparison) in gingival augmentation (Outcome)?

### 2.2. Search Strategy

The review was conducted following the Preferred Reporting Items for Systematic Reviews and Meta-Analyses (PRISMA2020) guidelines [36]. The conducted electronic literature search included the MEDLINE (PubMed), Embase, Google Scholar, and Scopus databases from inception until 28 June 2024. The keywords used in the searches of the Scopus, PubMed, and Google Scholar databases were (“microneedling” or “micro needling” or “injectable platelet-rich fibrin” or “i-PRF”)and (“gingival augmentation” or “augmentation” or “attached gingiva” or “attached mucosa” or “soft tissue augmentation” or “KM” or “keratinized mucosa”). The terms used in the Embase database search were (‘microneedling’/exp or microneedling or ‘micro needling’/exp or ‘micro needling’ or (micro and (‘needling’/exp or needling)) or ‘injectable platelet-rich fibrin’ or (injectable and ‘platelet rich’ and (‘fibrin’/exp or fibrin)) or ‘iprf’) and (‘gingival augmentation’ or (gingival and (‘augmentation’/exp or augmentation)) or ‘augmentation’/exp or augmentation or ‘attached gingiva’ or (attached and (‘gingiva’/exp or gingiva)) or ‘attached mucosa’ or (attached and (‘mucosa’/exp or mucosa)) or ‘soft tissue augmentation’/exp or ‘soft tissue augmentation’ OR (soft and (‘tissue’/exp or tissue) and (‘augmentation’/exp or augmentation)) OR km OR ‘keratinized mucosa’ or (keratinized and (‘mucosa’/exp or mucosa))). Furthermore, the authors conducted a “snowball” search to identify additional studies. This entailed a search of the bibliographies of the publications that had been deemed eligible for full-text review. Furthermore, Google Scholar was employed to identify and corroborate the veracity of the cited studies. An additional inclusion criterion restricted the electronic search to studies published in the English language. To minimize the risk of bias when searching for an article, the authors elected not to implement an electronic limitation in the form of randomized controlled trials. It is important to note that the categorization of academic papers is not always precise. Consequently, the most recent papers may not yet have been classified by the relevant criteria. The databases were searched by three authors (W.N., R.W., and J.Ż.), with the three sets of search terms being identical. Following the search process and the selection of potential studies for inclusion in the review, a joint assessment was conducted by all authors to ensure that the selected studies met all the inclusion criteria. To collate the data from the included studies, the two authors conducted a joint search of the literature to identify the desired data.

### 2.3. Selection of Studies

The objective of this systematic review was to evaluate the potential of gingival augmentation using i-PRF, either alone or in conjunction with microneedling. The criteria for the inclusion of articles in and exclusion of articles from this review are presented in Table 1.

### 2.4. Risk of Bias in Individual Studies

During the preliminary phase of the review selection process, each reviewer conducted a standalone assessment of titles and abstracts. This approach was adopted to minimize the influence of potential biases in the evaluation procedure. To quantify the level of inter-reviewer agreement, the authors employed a tool known as the Cohen’s к test [37]. In cases of discrepancy concerning the inclusion or exclusion of a study within the scope of the review, the respective authors deliberated until a consensus was reached.

### 2.5. Quality Assessment

Two independent reviewers, W.N. and R.W., conducted quality assessments of the included studies. The evaluation of the study design, implementation, and analysis was conducted in accordance with the following criteria: Random allocation of study participants (in the case of studies with a split-mouth study design, a random selection of quadrants); whether the study had a split-mouth study design; whether calculations were made before the study regarding the number of patients/study sites needed, and whether this criterion was met by the authors; balanced study/control groups within 10% of the participants or sites studied; whether it was clearly defined how the i-PRF was obtained from the patient and how it was administered, along with the number of sessions; whether it was specified how the gingival thickness was measured and whether tests of blood parameters with particular reference to platelet counts were performed prior to patient inclusion in the study. A total score of 3 points or less was indicative of a high risk of bias. A score of 4–6 points was indicative of a moderate risk of bias, while a score of 7 points and above represented a low risk of bias. Any discrepancies in scoring were resolved through discussion until a consensus was reached.

### 2.6. Risk of Bias across Studies

The scores for each study were calculated and the overall estimated risk of bias (low, moderate, high) was determined for each included study, following the recommendations set forth by the Cochrane Handbook for Systematic Reviews of Interventions [38]. All eight studies were assigned to the low risk of bias category. None of the included articles received the maximum score of nine. Six articles received eight points and two studies received seven points. No studies were excluded from the review based on low quality (high risk of bias), as the missing information was deemed not to be essential for the comprehensive assessment of the literature. A single point was allotted in the event of a positive response. Conversely, no additional points were assigned in instances of a negative or uncertain response. The risk of bias was assessed as low, moderate, or high. The exact level of bias in each included study is presented in Table 2.

### 2.7. Data Extraction

The following data were extracted from the articles included in the analysis, in addition to the risk of bias assessment. The country in which the study was conducted, the number of patients and study sites included in the study, the gender and age distribution of the study subjects, and the standard deviation were also recorded. The study groups, number of administrations of i-PRF, intervals between administrations, inclusion criterion based on gingival thickness, parameters assessed, results obtained, and follow-up period of patients were also considered. The results were synthesized in a narrative manner.

## 3. Results

### 3.1. Study Selection

**Figure 1:**
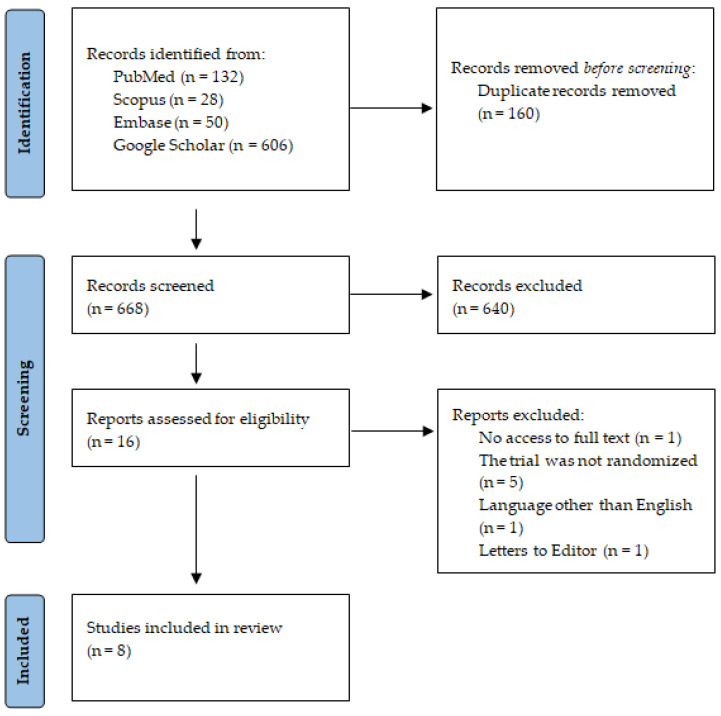
PRISMA 2020 flow diagram.

A flowchart illustrating the research methodology in accordance with the PRISMA statement [36] is presented in Figure 1. A search of the databases yielded 816 articles, of which 668 remained after the removal of duplicates. All of these were assessed based on the title, and some based on both the title and abstract. Of these, 16 remained. One study was excluded due to the unavailability of the full-text article [47], five were rejected due to the absence of randomization in the study [48,49,50,51,52], and one had only the title and abstract in English, while the remainder of the article was in another language [53]. One result was excluded as it was a letter to the editor [54]. The study comprised eight articles.

All articles included in the review were published within the last 5 years (2020–2024). All but one had a split-mouth design. Further details can be found in Table 3.

### 3.2. General Characteristics of the Included Studies

Of the eight articles, seven made a prior calculation of the required number of patients or study sites. The number of patients ranged from 10 to 36, and the number of study sites ranged from 40 to 360. All of the study authors utilized an endodontic spreader, number 15, and the distance of the apical part to the silicone disc was measured using a digital vernier caliper with a resolution of 0.01 mm. The collected data are categorized in Table 4.

The number of i-PRF injection sessions varied considerably between studies, from a single injection to four injections administered at 10-day intervals. Among the parameters studied in each study was gingival thickness, with seven out of eight studies also analyzing KTW. In addition to the parameters, gingival index, plaque index, pocket depth, bleeding on probing, pain experience using the visual analogue scale (VAS), and satisfaction with the aesthetic outcome were also evaluated. A summary of the key points from each article is presented in Table 5.

### 3.3. Main Study Outcomes

All included articles examined gingival thickness before the procedure and after a defined follow-up period, between three and six months. It could be seen that in all the studies there was a statistically significant increase in gingival thickness. However, the situation was different in the case of KTW. KTW was measured by seven of the eight studies included in the analysis, the only one that did not check this parameter was the Soundarajan and Malaippan study [45]. A statistically significant change in KTW was observed in the study by Valli Veluri et al. [39], Faour et al. [43], and Shakir and Salman [40], except of the upper arch. The articles under review compared i-PRF to a range of alternative treatments. A study by Valli Veluri et al. [39] compared the use of i-PRF to FGG and found no statistical difference between the groups, despite a significant increase in the gingival thickness parameter in both groups. A similar phenomenon was observed in the case of KTW. Nevertheless, the group undergoing non-surgical augmentation exhibited reduced post-surgical discomfort and greater satisfaction with the aesthetic result achieved. A study was conducted by Shakir and Salman [40], in which the efficacy of i-PRF was compared to that of c-PRF over threemonths. Injections were administered three times at weekly intervals. The primary findings indicated that c-PRF exhibited superior clinical outcomes in select instances, although these results did not reach statistical significance. The observed increase in gingival thickness and KTW was statistically significant in both cases. A study by Faour et al. [43] compared the efficacy of i-PRF with hyaluronic acid in non-surgical gingival augmentation. Both preparations were administered in three separate administrations, with each administration occurring at a weekly interval. A three-month follow-up demonstrated a significant increase in GT and KTW in both groups. Nevertheless, no significant differences were observed between the study groups in terms of the results. In addition, Manasa et al. [41] were the only study to compare i-PRF to a contralateral control group without any intervention. Nevertheless, the authors elected to administer only a single session of autologous i-PRF preparation. The results of the test group indicated a statistically significant increase in GT, with an overall increase of 26.56% after three months and 29% after six months in comparison to the baseline. No significant differences were observed in the width of the keratinized gingiva across any of the comparisons. The remaining half of the study was dedicated to a comparison of the effect of microneedling [42] or i-PRF [44,45,46], or to the additive effect of microneedling with i-PRF. Three studies were conducted with a six-month follow-up period, and intervention within groups was conducted four times at 10-day intervals. Soundarajan and Malaippan [45], however, conducted three sessions at 10-day intervals, with a follow-up period of three months. A study by Chetan et al. [42] demonstrated that the incorporation of i-PRF into MN leads to a statistically significant increase in gingival thickness in comparison to the MN-only group. This contrasts with the findings of the KTW measurements, where no statistically significant difference was observed between the two groups. In their study, Soundarajan and Malaippan [45] observed a significant increase in GT after the additional use of MN withi-PRF. Ozsagir et al. [46] demonstrated a significant increase in GT in both the i-PRF group and thei-PRF with MN group, whereas the use of i-PRF in conjunction with MN exhibited a significant statistical effect on KTW.

## 4. Discussion

The results of the articles reviewed demonstrated that the use of i-PRF has a statistically significant effect on gingival thickness [39,40,41,42,43,44,45,46]. Although the increase in GT does not appear to be substantial, it is important to consider that it is a minor yet impactful increase in fibroblast chemotaxis, which can consequently result in a stable and aesthetically appreciable increase in thickness [55]. The results regarding the effect of i-PRF use on KTW are not homogeneous. Some of the articles indicated a positive effect on this parameter [39,40,43], while others did not demonstrate statistical significance on this issue [41,42,44,46] or did not determine this parameter in the study [45]. The study by Valli Veluri et al. [39] was the only one to determine the patient’s subjective feelings in the form of their pain sensations and to compare satisfaction with the aesthetics of the procedure compared to FGG. In this context, the results demonstrated that i-PRF performed significantly better in terms of the parameters. Additionally, a non-randomized study by Fotani et al. investigating the effect of i-PRF on KTW demonstrated no statistically significant change in this parameter, while there was a significant, almost two-fold increase in gingival thickness [49]. Similar results were also shown in a study on five patients by Sonavane et al., where the mean increase of 0.62 mm in thickness was 42% [50]. In contrast, a non-randomized study by Tiwari et al. found that both GT and KTW demonstrated statistically significant increases in parameters [51]. A significant proportion of the included studies (50%) [42,44,45,46] addressed the topic of microneedling, also known as percutaneous collagen induction (PCI) [56]. The efficacy of PCI is contingent upon its ability to induce a controlled reparative process involving the coordinated development and remodeling of collagenous tissue through the release of growth factors. It has been demonstrated that the vascular endothelial growth factor (VEGF) and platelet-derived growth factor (PDGF) families play a crucial role in the healing process following needling [57]. Fibroblasts are responsible for the production of collagen and elastin fibers, which facilitate wound closure by migrating to the site of intrusion. Neocollagenesis is a process whereby newly formed fibers thicken the tissue. Additionally, fibroblasts facilitate neoangiogenesis by stimulating endothelial cell proliferation in vessels. The process of tissue remodeling can last for approximately eight weeks to a year [58,59,60]. Consequently, studies incorporating this procedure should be conducted over a longer follow-up period to ensure the reliability of the results. In addition, future research may consider the additional use of lasers, which can support fibroblast proliferation [61,62]. The Shakir and Salman study was the sole study conducted in comparison to c-PRF. Furthermore, it was the sole study in which the use of i-PRF yielded inferior outcomes when compared to the other study group. In 2019, Miron et al. demonstrated that c-PRF can concentrate platelets to a greater extent than i-PRF, with a concentration ratio of over 15 times above baseline. In contrast, the i-PRF procedure only concentrates platelets by approximately 2–3-fold. It would appear that this represents a significant advance in the field, as it represents the highest platelet and leukocyte concentration ever observed in any PRF preparation [63]. A significant limitation of this study is that a separate statistical analysis in the form of a meta-analysis was not conducted. This was a decision made by the authors of this article due to the lack of homogeneity between the articles included in the review. Most of the studies included different study groups, which could have introduced bias into the analysis, potentially influencing the objective assessment of the efficacy of i-PRF in gingival augmentation. Considering the limitations, the authors elected to present a comprehensive overview of all available randomized trials on the subject, thereby enabling future researchers to identify and highlight any pertinent aspects that may have been overlooked in the individual studies reviewed. However, the main limitation of this review is the small number of randomized trials. In the future, it will be necessary to carry out a systematic review and meta-analysis of a larger number of relevant articles. Although all included studies had a low risk of bias, the results presented are not without limitations. The analysis lacked multicenter trials to support the results presented. In future studies, the authors should also consider a longer follow-up period to determine the stability of the results obtained over time. It is noteworthy that none of the trials included in the analysis included blood tests as part of patient enrolment. Future authors should take this into account, as platelet counts can be significantly translated into platelet factors in blood-based treatments [64]. It is also important to consider the methodology employed to measure the thickness of the gums. While all of the included articles accurately presented the methodologies, none of the authors employed ultrasound measurements [65], which would have provided the results with the least potential for bias. All of the study authors utilized an endodontic spreader, number 15, and the distance of the apical part to the silicone disc was measured using a digital vernier caliper with a resolution of 0.01 mm. Nevertheless, the outcomes of the studies appear to be highly encouraging. Non-surgical augmentation may result in superior aesthetic outcomes, reduced postoperative discomfort for patients, and a lower risk of complications compared to surgical augmentation. This is due to the less invasive nature of the procedure, as well as the absence of two treatment sites, namely the donor and recipient site. This procedure may prove more straightforward for clinicians to perform than the surgical method. The option of non-surgical augmentation may prove to be a valuable addition to the orthodontic armamentarium for patients with the thin biotype, in whom the use of i-PRF may also facilitate tooth movement with braces [66,67,68]. Nevertheless, further randomized trials are required to enable a comparison of different configurations of the number of sessions with injections and the intervals between them.

## 5. Conclusions

This systematic review highlights the potential of injectable platelet-rich fibrin as an efficacious, non-surgical approach to gingival augmentation, particularly in patients with a thin gingival biotype. The analysis of eight randomized controlled trials revealed a significant increase in gingival thickness and, in some cases, an increase in keratinized tissue width following the use of i-PRF. The results of one study comparing i-PRF to FGG indicated that i-PRF yields outcomes that are comparable to those of traditional free gingival grafts, with the additional advantages of superior aesthetics and reduced postoperative discomfort. In light of these findings, i-PRF may represent a promising alternative in periodontal therapy, with the potential to inform the development of less invasive procedures for gingival augmentation in the future. Nevertheless, further research with larger sample sizes and longer follow-up periods is required to substantiate these promising findings and to establish standardized protocols for its clinical utilization.

## Figures and Tables

**Table 1:** Selection criteria for papers included in the systematic review.

Inclusion Criteria	Exclusion Criteria
Randomized controlled trials	Case reports/Case series
Full text available	Narrative reviews Systematic reviews
Human studies	Meta-analysis
English language	Non-English language publications
Patients aged ≥18 years	High risk of bias in the study
Non-smoking patients	Letters to editor Animal studies
Low or moderate risk of bias	Studies on smoking patients Conference papers

**Table 2:** The results of the quality assessment and risk of bias across the studies.

Study								
Criteria	Valli Veluri et al. (2024) [39]	Shakir and Salman (2023) [40]	Manasa et al. (2023) [41]	Chetana et al. (2024) [42]	Faour et al. (2022) [43]	Adhikary et al. (2023) [44]	Soundarajan and Malaippan (2023) [45]	Ozsagir et al. (2020) [46]
Random allocation	1	1	1	1	1	1	1	1
Split-mouth study type	0	1	1	1	1	1	1	1
Calculated study group	1	1	1	1	1	0	1	1
Balanced study groups	1	1	1	1	1	1	1	1
Clear method of obtaining i-PRF	1	1	1	1	1	1	1	1
Well defined method of i-PRF administration with number of sessions	1	1	1	1	1	1	1	1
Inclusion/exclusion criteria clearly defined	1	1	1	1	1	1	1	1
A clearly described methodology for measuring gingival thickness	1	1	1	1	1	1	1	1
Blood tests of patients prior to examination with platelet count assessment	0	0	0	0	0	0	0	0
Total	7	8	8	8	8	7	8	8
Risk of bias	Low	Low	Low	Low	Low	Low	Low	Low

**Table 3:** A general overview of the studies.

Author and Year	Country	Study Design	Split-Mouth
Valli Veluri et al. (2024) [39]	India	Randomized Controlled Clinical Trial	No
Shakir and Salman (2023) [40]	Iraq	Randomized Controlled Clinical Trial	Yes
Manasa et al. (2023) [41]	India	Randomized Controlled Clinical Trial	Yes
Chetana et al. (2024) [42]	India	Randomized Controlled Clinical Trial	Yes
Faour et al. (2022) [43]	Syria	Randomized Controlled Clinical Trial	Yes
Adhikary et al. (2023) [44]	India	Randomized Controlled Clinical Trial	Yes
Soundarajan and Malaippan (2023) [45]	India	Randomized Controlled Clinical Trial	Yes
Ozsagir et al. (2020) [46]	Turkey	Randomized Controlled Clinical Trial	Yes

**Table 4:** Characteristics of patients by study.

Author/Year	Sample Size Calculation	Number of Sites	Patients	Sex		Age (Years)	
				Female	Male	Mean (±SD)	Range
Valli Veluri et al. (2024) [39]	Yes	80	20	12	8	No data	26–48
Shakir and Salman (2023) [40]	Yes	40	10	No data		No data	
Manasa et al. (2023) [41]	Yes	360	30	24	6	28.53 ± 4.63	18–35
Chetana et al. (2024) [42]	Yes	120	15	6	9	26 ± 4	18–55
Faour et al. (2022) [43]	Yes	84	14	9	5	27.71 ± 7.47	18–40
Adhikary et al. (2023) [44]	No	64	32	No data		No data	18–34
Soundarajan and Malaippan (2023) [45]	Yes	216	36	23	13	32.4	20–45
Ozsagir et al. (2020) [46]	Yes	198	33	28	5	22.2	18–34

**Table 5:** Detailed characteristics of the studies included in this review.

Author/Year	Treatment Groups	Number of i-PRF Administration Sessions	Inclusion Criteria Based on GT	Evaluation	Main Results	Average GT Growth in mm	Follow-Up Period
Valli Veluri et al. (2024) [39]	1. FGG	4 sessions 10 days apart	GT < 0.8 mm	Pain in VAS Esthetic satisfaction GT KTW	Significant increase in GT and KTW with no intergroup variation (P = 0.32, 0.48, respectively) at the end of six months. i-PRF + MN group reduced discomfort (1.11 ± 0.60) and enhanced aesthetic satisfaction (8.77 ± 0.44).	1. 1.32	6 months
	2. i-PRF + MN					2. 1.25	
Shakir and Salman (2023) [40]	1. i-PRF	3 sessions 7 days apart	GT ≤ 1.0 mm	KTW GT	Significant difference at the three-month follow-up visit between groups. The mean difference between groups was ±1.373 mm, with an effect size of 0.2 at *p* = 0.048. The intra-group comparison was significant in both groups.	1. 0.3	3 months
	2. c-PRF					2. 0.37	
Manasa et al. (2023) [41]	1. i-PRF	1 session	Participants were categorized into thin (≤1 mm) and thick (≥1 mm)	GT KTW	A statistically significant increase in GT in the test group at both the individual site and tooth level. The test group exhibited an increase in GT of 26.56% after three months and 29% after six months, in comparison to the baseline. No significant differences were observed in the KTW in any of the comparisons.	1. 0.4	6 months
	2. Control group					2. 0.03	
Faour et al. (2022) [43]	1. i-PRF	3 sessions 7 days apart	GT ≤ 1.0 mm	GT KTW GI PD BOP	The GT increased significantly in both groups. The KTW also showed a statistically significant increase in the intragroup comparisons in both groups (*p* <0.05). No statistically significant difference was observed between the two groups at the three assessment times for the GT and the KTW (*p* >0.05).	1. 0.3	3 months
	2. Hyaluronic acid					2. 0.34	
Chetana et al. (2024) [42]	1. MN	4 sessions 10 days apart	GT < 1.5 mm	GTKTWGIPI	The mean GT at baseline in Group 1 was 0.454 ± 0.068 mm, while that of Group 2 was 0.451 ± 0.069 mm. This difference was not statistically significant. The mean GT was found to be significantly higher in Group 2 (0.647 ± 0.091 mm) vs. (0.566 ± 0.076 mm). There was no statistically significant difference in KTW observed in either group.	1. 0.11	6 months
	2. i-PRF + MN					2. 0.19	
Adhikary et al. (2023) [44]	1. i-PRF	4 sessions 10 days apart	GT < 0.8 mm	GTKTW	No significant difference in mean KTW between groups. The mean GT at six months and baseline was significantly higher in i-PRF with MN compared to i-PRF alone.	1. 0.12	6 months
	2. i-PRF + MN					2. 0.25	
Soundarajan and Malaippan (2023) [45]	1. i-PRF	3 sessions 10 days apart	GT < 0.8 mm	GT	A statistically significant difference between groups, with a greater increase in GT observed in the combination of MN and i-PRF in all the lower anterior regions.	1. 0.2	3 months
	2. i-PRF + MN					2. 0.23	
Ozsagir et al. (2020) [46]	1. i-PRF	4 sessions 10 days apart	GT < 0.8 mm	GT KTW	The second group showed a significantly greater increase in GT than the first group. In the intra-group comparisons, a statistically significant increase in GT was observed within both i-PRF [from 0.43 mm ± 0.14 to 0.62 mm ± 0.11 (*p*< 0.001)] and MN + i-PRF [from 0.4 mm ± 0.14 to 0.66 mm ± 0.12 (*p*< 0.001)] groups at the sixth month.Group 2 showed a statistically significant increase in KTW.	1. 0.19	6 months
	2. i-PRF + MN					2. 0.26	

FGG—free gingival graft;i-PRF—injectable platelet-rich fibrin; MN—microneedling; GT—gingival thickness; VAS—visual analogue scale; KTW—keratinized tissue width; PI—plaque index; GI—gingival index; c-PRF—concentrated platelet-rich fibrin; PD—probing depth; BOP—bleeding on probing.

## Data Availability

Not applicable.

## References

[1] Ochsenbein C., Ross S. (1969). A Reevaluation of Osseous Surgery. Dent. Clin. N. Am..

[2] Seibert J., Lindhe J. (1989). Esthetics and Periodontal Therapy. Textbook of Clinical Periodontology.

[3] Kao R.T., Pasquinelli K. (2002). Thick Versus Thin Gingival Tissue: A Key Determinant in Tissue Response to Disease and Restorative Treatment. J. Calif. Dent. Assoc..

[4] Abraham S., Deepak K.T., Ambili R., Preeja C., Archana V. (2014). Gingival Biotype and Its Clinical Significance—A Review. Saudi J. Dent. Res..

[5] Amid R., Kadkhodazadeh M., Moscowchi A., Tavakol Davani S., Soleimani M., Dehghani Soltani A., Al-Shuhayeb M. (2020). Effect of Gingival Biotype on Orthodontic Treatment-Induced Periodontal Complications: A Systematic Review. J. Adv. Periodontol. Implant Dent..

[6] Niemczyk W., Niemczyk S., Prokurat M., Grudnik K., Migas M., Wągrowska K., Lau K., Kasperczyk J. (2024). Etiology of Gingival Recession—A Literature Review. Wiad. Lek..

[7] Wennström J.L. (2014). Commentary: Treatment of Periodontitis: Effectively Managing Mucogingival Defects. J. Periodontol..

[8] Bednarz W., Żurek J., Gedrange T., Dominiak M. (2016). A Preliminary Clinical Comparison of the Use of Fascia Lata Allograft and Autogenous Connective Tissue Graft in Multiple Gingival Recession Coverage Based on the Tunnel Technique. Adv. Clin. Exp. Med..

[9] Zweers J., Thomas R.Z., Slot D.E., Weisgold A.S., Van Der Weijden F.G.A. (2014). Characteristics of Periodontal Biotype, Its Dimensions, Associations and Prevalence: A Systematic Review. J. Clin. Periodontol..

[10] Agarwal V., Sunny, Mehrotra N., Vijay V. (2017). Gingival Biotype Assessment: Variations in Gingival Thickness with Regard to Age, Gender, and Arch Location. Indian J. Dent. Sci..

[11] Malpartida-Carrillo V., Tinedo-Lopez P.L., Guerrero M.E., Amaya-Pajares S.P., Özcan M., Rösing C.K. (2021). Periodontal Phenotype: A Review of Historical and Current Classifications Evaluating Different Methods and Characteristics. J. Esthet. Restor. Dent..

[12] Cook D.R., Mealey B.L., Verrett R., Mills M., Noujeim M., Lasho D., Cronin R.J. (2011). Relationship between Clinical Periodontal Biotype and Labial Plate Thickness: An in Vivo Study. Int. J. Periodontics Restor. Dent..

[13] Kan J., Morimoto T., Rungcharassaeng K., Roe P., Smith D.H. (2016). Gingival Biotype Assessment in the Esthetic Zone: Visual versus Direct Measurement. Int. J. Periodontics Restor. Dent..

[14] Shah H.K., Sharma S., Shrestha S. (2020). Gingival Biotype Classification, Assessment, and Clinical Importance: A Review. J. Nepal. Soc. Periodontol. Oral Implantol..

[15] Barootchi S., Tavelli L., Zucchelli G., Giannobile W.V., Wang H. (2020). Gingival Phenotype Modification Therapies on Natural Teeth: A Network Meta-analysis. J. Periodontol..

[16] Kim D.M., Neiva R. (2015). Periodontal Soft Tissue Non–Root Coverage Procedures: A Systematic Review From the AAP Regeneration Workshop. J. Periodontol..

[17] Fernandes G.D.O., Santos N.M., Siqueira R.C.D., Wang H.-L., Blanco-Carrion J., Fernandes J.H. (2021). Autologous Platelet Concentrate of 2nd and 3rd Generations Efficacy in the Surgical Treatment of Gingival Recession: An Overview of Systematic Reviews. J. Indian Soc. Periodontol..

[18] Niemczyk W., Janik K., Żurek J., Skaba D., Wiench R. (2024). Platelet-Rich Plasma and Injectable Platelet-Rich Fibrin (i-PRF) in the Non-Surgical Treatment of Periodontitis—A Systematic Review. Int. J. Mol. Sci..

[19] Prokurat M., Grudnik K., Niemczyk W., Niemczyk S., Migas M., Wągrowska K., Lau K., Kasperczyk J. (2024). Platelet-Rich Plasma—A Remedy Present in Every Human Being. History, Functioning, and the Benefits of Therapy Using It. Pol. Merkur. Lek..

[20] Miron R.J., Zucchelli G., Pikos M.A., Salama M., Lee S., Guillemette V., Fujioka-Kobayashi M., Bishara M., Zhang Y., Wang H.-L. (2017). Use of Platelet-Rich Fibrin in Regenerative Dentistry: A Systematic Review. Clin. Oral Investig..

[21] Saini K., Chopra P., Sheokand V. (2020). Journey of Platelet Concentrates: A Review. Biomed. Pharmacol. J..

[22] Tang B., Huang Z., Zheng X. (2023). Impact of Autologous Platelet Concentrates on Wound Area Reduction: A Meta-analysis of Randomized Controlled Trials. Int. Wound J..

[23] Chou T., Chang H., Wang J. (2020). Autologous Platelet Concentrates in Maxillofacial Regenerative Therapy. Kaohsiung J. Med. Sci..

[24] Farshidfar N., Jafarpour D., Firoozi P., Sahmeddini S., Hamedani S., De Souza R.F., Tayebi L. (2022). The Application of Injectable Platelet-Rich Fibrin in Regenerative Dentistry: A Systematic Scoping Review of In Vitro and In Vivo Studies. Jpn. Dent. Sci. Rev..

[25] Miron R.J., Fujioka-Kobayashi M., Hernandez M., Kandalam U., Zhang Y., Ghanaati S., Choukroun J. (2017). Injectable Platelet Rich Fibrin (i-PRF): Opportunities in Regenerative Dentistry?. Clin. Oral Investig..

[26] Dominiak M., Mierzwa-Dudek D., Konopka T., Całka K. (2003). Zagęszczona Masa Płytek Krwi (PRP) w Leczeniu Recesji Dziąsła—Badania Wstępne. Dent. Med. Probl..

[27] Karde P., Sethi K., Mahale S., Khedkar S., Patil A., Joshi C. (2017). Comparative Evaluation of Platelet Count and Antimicrobial Efficacy of Injectable Platelet-Rich Fibrin with Other Platelet Concentrates: An in Vitro Study. J. Indian Soc. Periodontol..

[28] Deepali B. (2012). Collagen Induction Therapy With Dermaroller. Community Based Med. J..

[29] Choukroun J., Ghanaati S. (2018). Reduction of Relative Centrifugation Force within Injectable Platelet-Rich-Fibrin (PRF) Concentrates Advances Patients’ Own Inflammatory Cells, Platelets and Growth Factors: The First Introduction to the Low Speed Centrifugation Concept. Eur. J. Trauma Emerg. Surg..

[30] Crisci A., Crescenzo U.D., Crisci M. (2018). Platelet-Rich Concentrates (L-PRF, PRP) in Tissue Regeneration: Control of Apoptosis and Interactions with Regenerative Cells. J. Clin. Mol. Med..

[31] Cieslik-Bielecka A., Ehrenfest D.M.D., Lubkowska A., Bielecki T. (2012). Microbicidal properties of leukocyte-and platelet-rich plasma/fibrin (L-PRP/L-PRF): New perspectives. J. Biol. Regul. Homeost. Agents.

[32] Varshney S., Dwivedi A., Pandey V. (2019). Antimicrobial Effects of Various Platelet Rich Concentrates-Vibes from in-Vitro Studies-a Systematic Review. J. Oral Biol. Craniofacial Res..

[33] Gollapudi M., Bajaj P., Oza R.R. (2022). Injectable Platelet-Rich Fibrin—A Revolution in Periodontal Regeneration. Cureus.

[34] Varela H.A., Souza J.C.M., Nascimento R.M., Araújo R.F., Vasconcelos R.C., Cavalcante R.S., Guedes P.M., Araújo A.A. (2019). Injectable Platelet Rich Fibrin: Cell Content, Morphological, and Protein Characterization. Clin. Oral Investig..

[35] Schardt C., Adams M.B., Owens T., Keitz S., Fontelo P. (2007). Utilization of the PICO Framework to Improve Searching PubMed for Clinical Questions. BMC Med. Inform. Decis. Mak..

[36] Page M.J., McKenzie J.E., Bossuyt P.M., Boutron I., Hoffmann T.C., Mulrow C.D., Shamseer L., Tetzlaff J.M., Akl E.A., Brennan S.E. (2021). The PRISMA 2020 Statement: An Updated Guideline for Reporting Systematic Reviews. BMJ.

[37] Watson P.F., Petrie A. (2010). Method Agreement Analysis: A Review of Correct Methodology. Theriogenology.

[38] Higgins J., Thomas J., Chandler J., Cumpston M., Li T., Page M. (2023). Welch Cochrane Handbook for Systematic Reviews of Interventions Version 6.4.

[39] Valli Veluri S., Gottumukkala S.N., Penmetsa G.S., Ramesh K., Bypalli V., Vundavalli S., Gera D. (2024). Clinical and Patient-Reported Outcomes of Periodontal Phenotype Modification Therapy Using Injectable Platelet Rich Fibrin with Microneedling and Free Gingival Grafts: A Prospective Clinical Trial. J. Stomatol. Oral Maxillofac. Surg..

[40] Shakir S.A., Salman S.A. (2023). Efficacy of Concentrated Platelets-Rich Fibrin versus Injectable Platelets Rich Fibrin on Gingival Thickness and Keratinized Tissue Width in Subjects with Thin Gingival Phenotype: Split-Mouth Randomized Clinical Trial. Al-Rafidain J. Med. Sci..

[41] Manasa B., Baiju K.V., Ambili R. (2023). Efficacy of Injectable Platelet-Rich Fibrin (i-PRF) for Gingival Phenotype Modification: A Split-Mouth Randomized Controlled Clinical Trial. Clin. Oral Investig..

[42] Chetana, Sidharthan S., Dharmarajan G., Iyer S., Poulose M., Guruprasad M., Chordia D. (2024). Evaluation of Microneedling with and without Injectable-Platelet Rich Fibrin for Gingival Augmentation in Thin Gingival Phenotype-A Randomized Clinical Trial. J. Oral Biol. Craniofacial Res..

[43] Faour N.H., Dayoub S., Hajeer M.Y. (2022). Evaluation of the Hyaluronic Acid Versus the Injectable Platelet-Rich Fibrin in the Management of the Thin Gingival Phenotype: A Split-Mouth Randomized Controlled Clinical Trial. Cureus.

[44] Adhikary R., Mohan P., Wadhawan A., Tyagi P. (2023). Gingival Augmentation in the Thin Phenotype Using Injectable Platelet-Rich Fibrin and Microneedling. Cureus.

[45] Soundarajan S., Malaippan S. (2023). Injectable Platelet-Rich Fibrin and Microneedling—A Non-Surgical Approach for Gingival Augmentation: A Randomized Controlled Clinical Trial. J. Adv. Oral Res..

[46] Ozsagir Z.B., Saglam E., Sen Yilmaz B., Choukroun J., Tunali M. (2020). Injectable Platelet-rich Fibrin and Microneedling for Gingival Augmentation in Thin Periodontal Phenotype: A Randomized Controlled Clinical Trial. J. Clin. Periodontol..

[47] Yadav A., Tanwar N., Sharma R., Tewari S. (2024). Aditi Sangwan Comparative Evaluation of Microneedling vs Injectable Platelet-Rich Fibrin in Thin Periodontal Phenotype: A Split-Mouth Clinical Randomized Controlled Trial. Quintessence Int..

[48] Alan R., Ercan E., Firatli Y., Firatli E., Tunali M. (2023). Innovative I-PRF Semisurgical Method for Gingival Augmentation and Root Coverage in Thin Periodontal Phenotypes: A Preliminary Study. Quintessence Int..

[49] Fotani S., Shiggaon L.B., Waghmare A., Kulkarni G., Agrawal A., Tekwani R. (2019). Effect of Injectable Platelet Rich Fibrin (i-PRF) on Thin Gingival Biotype: A Clinical Trial. J. Appl. Dent. Med. Sci..

[50] Sonavane P.A., Rai J.J., Shah M.A., Andharia M.S. (2023). Effect of Microneedling and Injectable Platelet-Rich Fibrin on Gingival Phenotype—A Case Series. J. Datta Meghe Inst. Med. Sci. Univ..

[51] Tiwari V., Agarwal S., Goswami V., Gupta B., Khiraiya N., Soni V.R. (2022). Effect on Injectable Platelet Rich Fibrin in Augmentation of Thin Gingival Biotype: A Clinical Trial. Int. J. Health Sci..

[52] Akolu P., Lele P., Dodwad V., Khot T., Kundoo A., Bhosale N. (2023). Comparative evaluation of the effect of injectable platelet rich fibrin (I-PRF) on gingival thickness in individuals with thin periodontal phenotype—A clinical STUDY. Eur. Chem. Bull..

[53] Sağlam E., Özsağir Z.B., Ünver T., Toprak A., Bayer Alinca S., Tunali M. (2020). EROZİV ORAL LİKEN PLANUS’TA ENJEKTE EDİLEBİLEN TROMBOSİTTEN ZENGİN FİBRİN: ÇİFT KÖR, BÖLÜNMÜŞ AĞIZ, RANDOMİZE KONTROLLÜ PİLOT ÇALIŞMA. SDÜ Tıp Fakültesi Dergisi.

[54] Gurgel B.V., Juliasse L., Pinto N., Ghanaati S., Mourão C.F. (2024). RE: “Evaluation of Microneedling with and without Injectable-Platelet Rich Fibrin for Gingival Augmentation in Thin Gingival Phenotype—A Randomized Clinical Trial”. J. Oral Biol. Craniofacial Res..

[55] Vesala A.-M., Nacopoulos C., Gkouskou K., Amenta F., Ruga E. (2021). Microneedling with Injectable Platelet-Rich Fibrin for Facial Rejuvenation. Plast. Aesthetic Res..

[56] Fernandes D. (2005). Minimally Invasive Percutaneous Collagen Induction. Oral Maxillofac. Surg. Clin. N. Am..

[57] El-Domyati M., Barakat M., Awad S., Medhat W., El-Fakahany H., Farag H. (2015). Microneedling Therapy for Atrophic Acne Scars. J. Clin. Aesthetic Dermatol..

[58] McCormick S.A. (2012). Induction of Dermal Collagenesis, Angiogenesis, and Adipogenesis in Human Skin by Injection of Platelet-Rich Fibrin Matrix. Arch. Facial Plast. Surg..

[59] Sterczała B., Chwiłkowska A., Szwedowicz U., Kobielarz M., Chwiłkowski B., Dominiak M. (2022). Impact of APRF+ in Combination with Autogenous Fibroblasts on Release Growth Factors, Collagen, and Proliferation and Migration of Gingival Fibroblasts: An In Vitro Study. Materials.

[60] Smith P.C., Martínez C., Martínez J., McCulloch C.A. (2019). Role of Fibroblast Populations in Periodontal Wound Healing and Tissue Remodeling. Front. Physiol..

[61] Sterczała B., Grzech-Leśniak K., Michel O., Trzeciakowski W., Dominiak M., Jurczyszyn K. (2021). Assessment of Human Gingival Fibroblast Proliferation after Laser Stimulation In Vitro Using Different Laser Types and Wavelengths (1064, *980*, 635, 450, and 405 Nm)—Preliminary Report. J. Pers. Med..

[62] Motlagh K.H., Azizi A., Ghadim N.M. (2023). In Vitro Effect of 445 Nm Blue Laser and 660 Nm LOW-LEVEL Laser on the Quantity and Quality of Human Gingival Fibroblasts. Photochem. Photobiol..

[63] Miron R.J., Chai J., Zhang P., Li Y., Wang Y., Mourão C.F.D.A.B., Sculean A., Fujioka Kobayashi M., Zhang Y. (2020). A Novel Method for Harvesting Concentrated Platelet-Rich Fibrin (C-PRF) with a 10-Fold Increase in Platelet and Leukocyte Yields. Clin. Oral Investig..

[64] Andrade M., Defreitasbrandao C., Sa C., Debittencourt T., Sadigursky M. (2008). Evaluation of Factors That Can Modify Platelet-Rich Plasma Properties. Oral Surg. Oral Med. Oral Pathol. Oral Radiol. Endodontol..

[65] Puzio M., Błaszczyszyn A., Hadzik J., Dominiak M. (2018). Ultrasound Assessment of Soft Tissue Augmentation around Implants in the Aesthetic Zone Using a Connective Tissue Graft and Xenogeneic Collagen Matrix—1-Year Randomised Follow-Up. Ann. Anat. Anat. Anz..

[66] Farshidfar N., Amiri M.A., Firoozi P., Hamedani S., Ajami S., Tayebi L. (2022). The Adjunctive Effect of Autologous Platelet Concentrates on Orthodontic Tooth Movement: A Systematic Review and Meta-Analysis of Current Randomized Controlled Trials. Int. Orthod..

[67] Erdur E.A., Karakaslı K., Oncu E., Ozturk B., Hakkı S. (2021). Effect of Injectable Platelet-Rich Fibrin (i-PRF) on the Rate of Tooth Movement. Angle Orthod..

[68] Zeitounlouian T.S., Zeno K.G., Brad B.A., Haddad R.A. (2021). Three-Dimensional Evaluation of the Effects of Injectable Platelet Rich Fibrin (i-PRF) on Alveolar Bone and Root Length during Orthodontic Treatment: A Randomized Split Mouth Trial. BMC Oral Health.

